# Corrigendum to: Beneficial Effects of *Lacticaseibacillus paracasei* subsp. *paracasei* ABF21013 (ACE-01) on Skin Health by Regulating MMPs Production and EGFR Signal Pathway

**DOI:** 10.4014/jmb.2026.3604.C04

**Published:** 2026-06-12

**Authors:** Sol Lee, Eun Jeoung Lee, Chang won Ahn, Han-Oh Park, Changmin Lee

**Affiliations:** 1 AceBiome Inc., Seoul 06164, Republic of Korea; 2 R&D Center, AceBiome Inc., Daejeon 34013, Republic of Korea; 3 Bioneer Corporation, Daejeon 34302, Republic of Korea

In the article titled “Beneficial Effects of *Lacticaseibacillus paracasei* subsp. *paracasei* ABF21013 on Skin Health by Regulating MMPs Production and EGFR Signal Pathway, we noticed a typographical error in Fig. S3 of the Supplementary Data. We would therefore like to revise Fig. S3 and submit the corrected version.

We would like to correct two typographical errors in Fig. S3 and its legend. In the legend of Fig. S3, “HT-92 cells” should be revised to “HT-29 cells.” In addition, the strain number indicated in the figure should be corrected from “ABF21069” to “ABF21013.”

We clarified that ABF21013 and ACE-01 refer to the same strain name and share the same deposit/accession number [deposit number: KCTC 16093BP].

We acknowledge these corrections and confirm that they do not affect the results, interpretations, or conclusions of the article.[Fig F1][Fig F2][Fig F3][Fig F4][Fig F5]

## Figures and Tables

**Fig. 1 F1:**
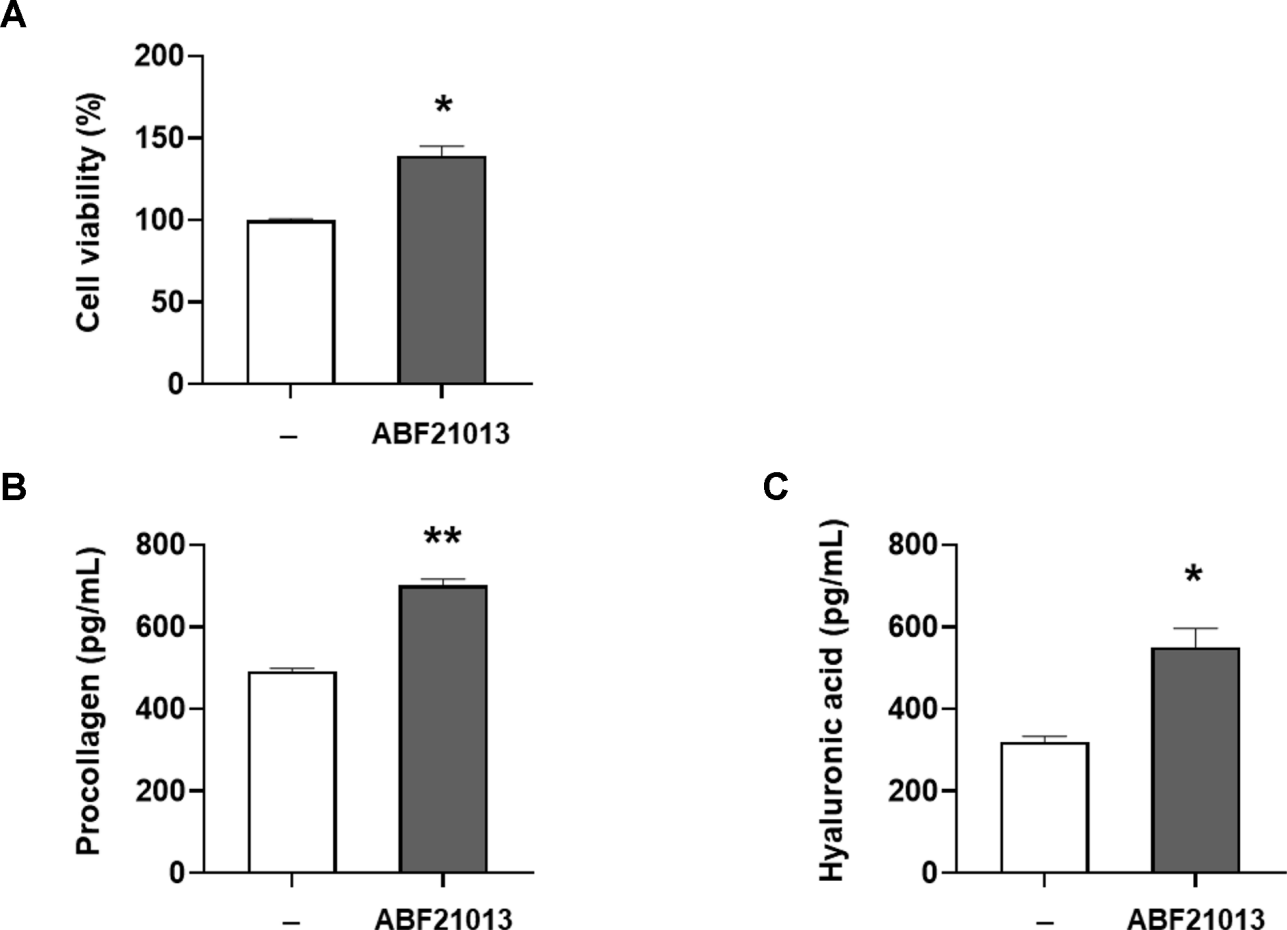
Fig. S1. Effects of cell-free supernatant from ABF21013 (CFS) on (A) cell viability (B) procollagen production, and (C) hyaluronic acid production in Hs68 cells. Data are two independent experiments. * *p* < 0.05 vs. NC group

**Fig. 2 F2:**
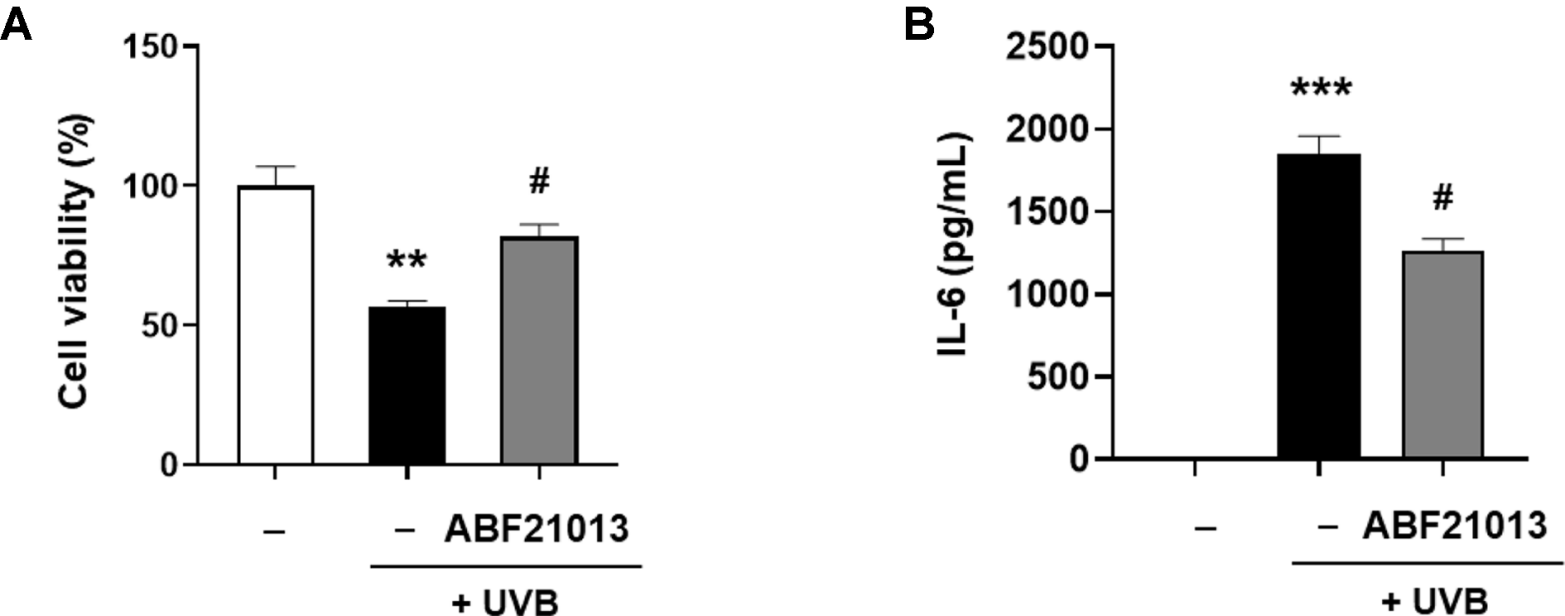
Fig. S2. Effects of CFS on (A) UVB-induced damage protection and (B) anti-inflammatory effect for IL-6 in UVB-irradiated HaCaT cells. Data are two independent experiments. ** *p* < 0.01, *** *p* < 0.001 vs. NC group; # *p*< 0.05 vs. UVB group

**Fig. 3 F3:**
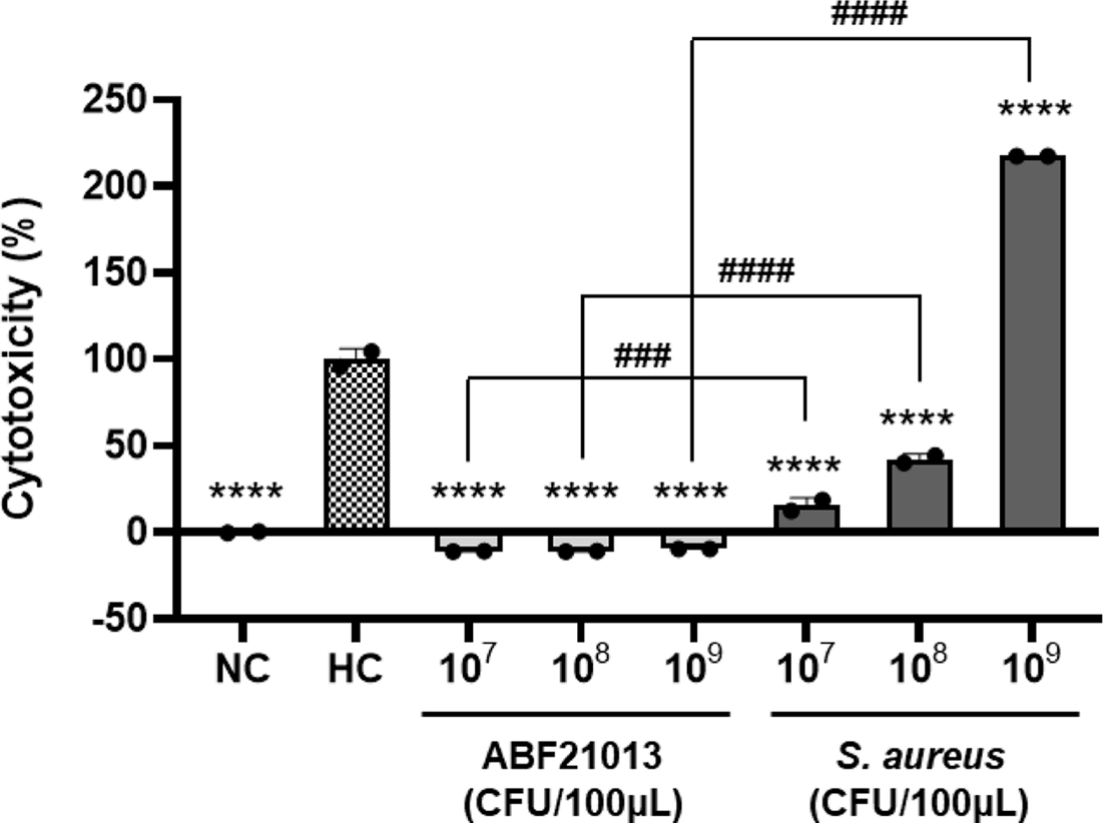
Fig. S3. Cytotoxicity of the ABF21013 against HT-29 cells. LDH assay performed on HT-29 cells after ABF21013 exposure to estimate their cytotoxicity. Data are duplicated experiments. NC, negative control; HC, positive control in cells were damaged with lysate solution; *S. aureus*, positive control in which cells were damaged with *S. aureus*. **** *p*< 0.0001 vs. HC group; ### *p*<0.001, #### *p*<0.0001 vs *S. aureus* group.

**Fig. 4 F4:**
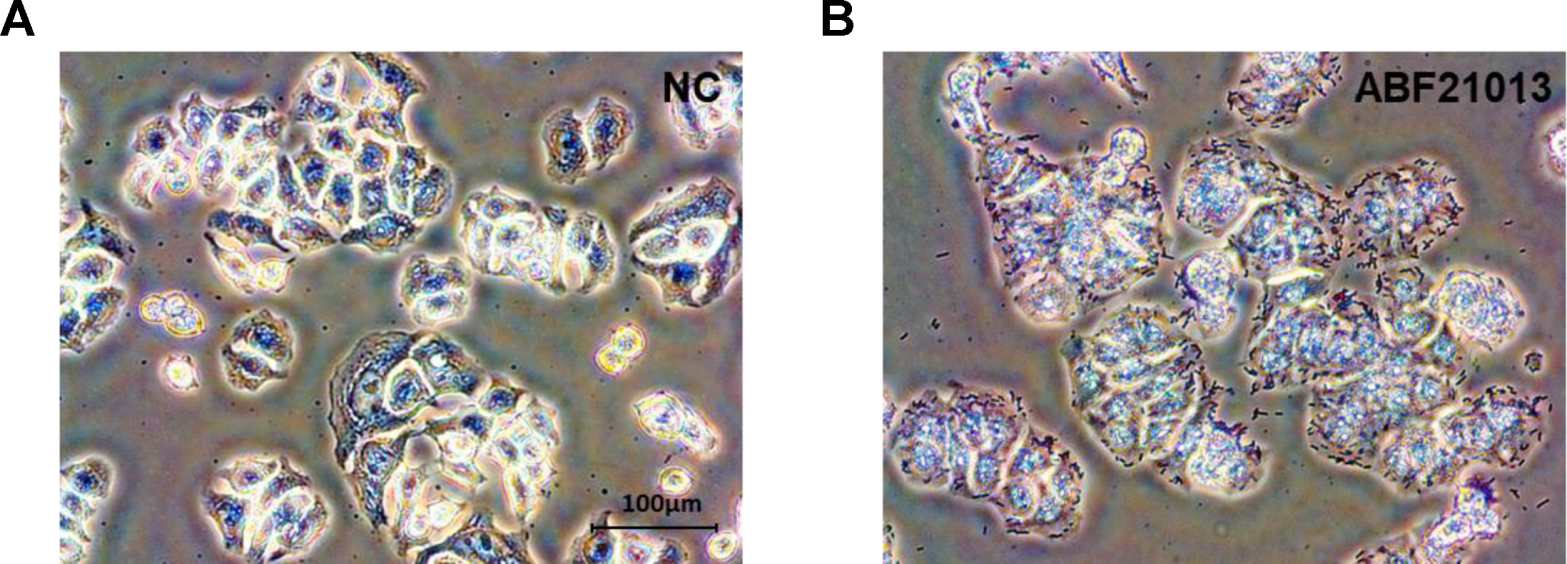
Fig. S4. Adhesion of ABF21013 to HT-29 cells. (**A**) Untreated HT-29 cells, (**B**) ABF21013. Data are representative of two independent experiments.

**Fig. 5 F5:**
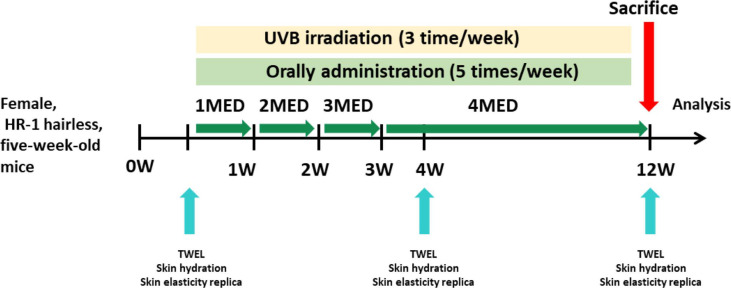
Fig. S5. Experimental Design. Study design of UVB-induced hairless mouse model and administration of ABF21013.

